# Advances in the Structural Strategies of the Self-Assembly of Photoresponsive Supramolecular Systems

**DOI:** 10.3390/ijms23147998

**Published:** 2022-07-20

**Authors:** Vivian J. Santamaria-Garcia, Domingo R. Flores-Hernandez, Flavio F. Contreras-Torres, Rodrigo Cué-Sampedro, José Antonio Sánchez-Fernández

**Affiliations:** 1 Escuela de Ingeniería y Ciencias, Tecnologico de Monterrey, Campus Monterrey, Avenida Eugenio Garza Sada 2501, Monterrey 64849, Mexico; visantamaria@tec.mx (V.J.S.-G.); drflores@tec.mx (D.R.F.-H.); contreras.flavio@tec.mx (F.F.C.-T.); rodrigo.cue.sampedro@tec.mx (R.C.-S.); 2 Procesos de Polimerización, Centro de Investigación en Química Aplicada, Blvd. Enrique Reyna No. 140, Saltillo 25294, Mexico

**Keywords:** photoresponsive, supramolecular structures, photoswitchable systems, azobenzene, diethienylehene, arylazopyrazole, metal–organic cages, metal–organic frameworks, hydrogen-bonded organic frameworks

## Abstract

Photosensitive supramolecular systems have garnered attention due to their potential to catalyze highly specific tasks through structural changes triggered by a light stimulus. The tunability of their chemical structure and charge transfer properties provides opportunities for designing and developing smart materials for multidisciplinary applications. This review focuses on the approaches reported in the literature for tailoring properties of the photosensitive supramolecular systems, including MOFs, MOPs, and HOFs. We discuss relevant aspects regarding their chemical structure, action mechanisms, design principles, applications, and future perspectives.

## 1. Introduction

Nanoscience and its related fields have the potential to lead humanity to the understanding and mastery of learning the highly efficient design principles found in nature to resolve a plethora of current technological challenges. Stimuli-responsive materials have attracted significant attention in material science. In response to external stimuli, they can undergo substantial micro- or nano-scale changes with exquisite spatial and temporal control, thus they are considered “smart materials”. The stimuli disturbing these materials are of a different nature. Among the most studied are those with a chemical nature, such as pH [1], ionic solvent [2], and chemical agents [3], and others with a physical nature, such as temperature, mechanical force, light [4,5], and electric or magnetic fields [6]. However, biochemical stimuli are also frequently applied [7]. The wide practical application of smart materials involves sensing, drug delivery, additive manufacturing, and photochromic devices [8].

Light is a worthy ubiquitous stimulus whose intensity and wavelength can be easily controlled according to application requirements. The light-triggered response of a photosensitive system may be reversible or not. However, it has been proved that the reversible responses allow the system to shift, commonly between two states that provide a way to switch the system on or off and hence to catalyze and control a reaction. Most of the reversible photo-induced reactions are described by the photochromism phenomenon. This involves a reversible toggling between two isomers of the photochromic molecule, i.e., reversible photoisomerization [9,10]. Similar to other photochemical reactions, photochromism corresponds to an equilibrium state; thus, the product does not appear as a separate phase but results as distinguishable due to the reversibility attribute. Each of the two states exhibits a characteristic absorption spectrum that results from electromagnetic radiation in at least one direction.

Azobenzene is a well-studied photochromic unit that governs the photoisomerization processes reported in the literature. It is a *T*-type of a photochrome that shows the capability of going from an *E*(*trans*) to a *Z*(*cis*) conformation when being irradiated with ultraviolet (UV) light, which exerts structural stresses on the molecule [11,12]. Some of the structural changes imply modifications in bonding angles, dipole moments, and even conjugation properties [13]. Moieties such as azobenzenes have commonly enabled highly efficient transformations in the forward and reverse directions by altering their steric and electronic properties [14].

Photoswitchable systems designed to catalyze chemical reactions must incorporate the appropriate photochromic units into the system to translate the structural switching states into a different chemical reactivity [9]. Therefore, one must optimize the intrinsic switching properties of the photochrome in a photoisomerization process to attend to a specific target task by activating or deactivating an initial material, the product, or a dye mediator [15,16]. The vast majority of the photoswitchable systems fall into one of the following categories, which will be discussed later: metal–organic cages (MOCs), metal–organic frameworks, or hydrogen-bonded organic frameworks (HOFs).

Metal–ligand interactions have been exploited for the construction of photoswitchable cages and frameworks. The coordination of organic ligands to metal ions gives rise to well-defined cavities that confer functional properties to modulate reactions in a very similar fashion to enzymes. On the other hand, hydrogen bonding is a donor–acceptor interaction specifically involving hydrogen atoms interacting with an acceptor carrying nonbonding electron lone pairs. Hydrogen bonding is an essential component of the structure and function of biological molecules since they highly influence molecular packing and solvation, and even alter conformation. They can serve as molecular interactions that control and stabilize the supramolecular organization through self-assembly, thus directly impacting functionality aspects [10]. A key factor in the formation of hierarchical structures with asymmetrical and/or anisotropic organization emerges in nanoarchitectonics-based material constructions, such as in most biological functional systems or bio-like highly functional materials, which are fundamental for creating rational flows of energy and efficient photocatalysts [17,18].

To overcome the current limitations regarding the low chemical and thermal stability of the photosensitive systems, the incorporation of dyes is one of the adopted strategies. The combination of dyes into metal–organic systems has the purpose of assisting the light-dependent mechanism either as an organic ligand, metal node, or as a cavity encapsulated guest. However, new methods of materials synthesis must be developed in order to realize the next generation of photoreactive materials through optical ways that take advantage of the involving electron transfer mechanisms [19,20].

This review focuses on the structure of supramolecular systems with photoresponsive units and their action mechanisms, highlighting some of the applied design principles and applications. We review the approaches toward achieving the photoswitchable control of supramolecular organic systems with a dye incorporated into metal–organic cages (MOCs) and metal–organic frameworks (MOFs). We also discuss the azobenzene and diethienylethene bearing photoswitchable catalysts. Finally, we outline the current challenges and possible directions for future research on this promising class of light-gated systems.

## 2. Supramolecular Structures with Photoresponsive Units

Substantial advances in materials science would not have been possible without the application and understanding of chemistry. The impact of chemistry greatly agglutinates to the transformation and control of the use of electromagnetism on photoreactions and photochromism phenomena, creating countless possibilities for developing new photoswitchable systems. The coupling of photosensitive systems to nanoparticles is a convenient route for the design of specialized materials. It is well known that most of the relevant properties of nanoparticles depend on their size and morphology. For example, for nanomedicine applications, particles must have dimensions of 10–100 nm for optimum circulation in the bloodstream and to avoid renal and lymphatic clearance. In this regard, Manners and co-workers explored the possibilities of creating π conjugated polymer nanoparticles sensitive to light and focused on self-assembly methods [21]. The reported strategies conceptualize a photoresponsive, spiropyran-coated, nanostructured surface that enables the highly efficient release of cancer cells [22]. Great progress in the photoresponsive system for in vivo application might be achieved by exploring the self-illuminating induced photocleavage of linkers based on abnormal disease microenvironments [23].

In the quest to attain the best performance of carbazole moieties in carbazole-based copolymers (CPs) in organic field-effect transistor (OFET) memory devices, a highly valued aspect is the search for the best possible conjugation state. The electronic polarization of the carbazole ring enhances the dipole−dipole interactions [24]. The study of the structure−property relationship of the carbazole-based copolymers has shown the relation of the donor−acceptor system with the modulation of the performance of photoresponsive OFET memory devices [25]. In response to these findings, Ono et al. postulated the integration of chiral moieties into carbazole-cored molecules to enhance the photosensitive material properties [26].

Experiments with various donor–acceptor-type CPs such as poly(1,8-carbazole)-benzothiadiazole copolymer (PCzBT) and a poly(1,8-carbazole)-dithienylbenzothiadiazole copolymer (PCzDTBT) with high photostability as well as diameters as small as 3.0–4.5 nm were described by Piwonski et al. [27]. Under the optimal biological regulatory mechanisms, a variety of reversible assembly/disassembly strategies for targeting switchable photocatalytic systems may be successfully extended for supramolecular designs that can facilitate nonparallel stacking of electron-donor (D) and electron-acceptor (A) moieties in constructing indistinguishable photoredox catalytic systems [28,29]. The phenomenon of an intramolecular charge-transfer interaction between an electron-rich carbazole fragment and an electron-deficient iminium double bond leads to a broad absorption in the visible spectrum. A charge transfer complex can be irradiated to trigger a single electron transfer process from the carbazole to the iminium ion, developing a chiral radical intermediate [28].

Biomimetics may be combined with engineering techniques to create bioengineered materials against bacterial infections. An interesting approach is the conjugation of molecules or proteins to natural or semisynthetic polymers, developing bioengineered polymer–drug conjugates [30]. In the same line, several of the architectonic characteristics of biomolecules, in particular nucleic acids, proteins, and sugars, that use diverse chemical interactions to form well-defined architectures with distinctive functions have a clear advantage because of their self-assembling properties [31]. The importance of self-assemble colloidal crystals requires a deeper scientific understanding of ligand–particle and particle–fluid interactions at multiple scales and governed by various conditions according to their nature [32].

**Scheme 1:**
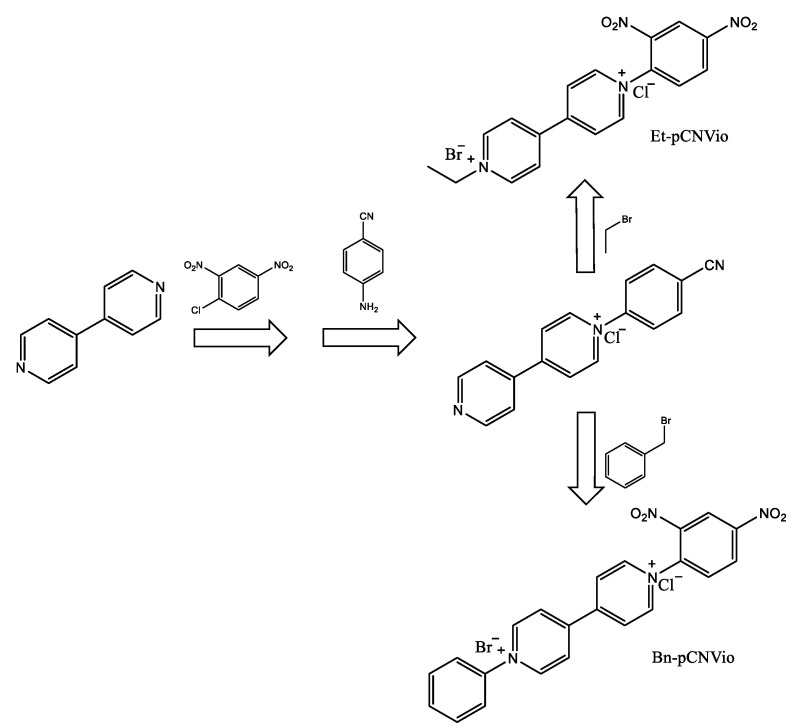
Synthesis route to obtain the asymmetric viologens Et*p*CNVio and Bn-*p*CNVio [33].

Photoactive molecules such as viologenic compounds can be found within organic electrochromic materials in different states. The three common redox forms of viologens are the usually colorless di-cation, the highly colored radical cation, and the di-reduced (neutral) form. The electrochromic properties of the viologen systems can be modulated by varying the molecular structure by changing the substituents on the nitrogen atom and by changing their environment (electrolytic media). Electrochromic (EC) gels based on single asymmetric viologens provide a more neutral-colored state than their corresponding symmetric viologens while maintaining a satisfactory colorless, bleached state and optical contrasts. The authors of such findings worked with two asymmetrically 1-alkyl-1′-aryl-substituted viologens, specifically 1-ethyl-1′-(*p*-cyanophenyl)-4,4′-bipyridinium dibromide (Et-*p*CNVio) and 1-benzyl-1′-(*p*-cyanophenyl)-4,4′-bipyridinium dibromide (Bn-*p*CNVio). Electrochromic devices (ECDs) based on alkyl substituents show violet coloration, while viologens with aryl groups demonstrate green coloration for their first reduced form [33]. The synthetic route successfully followed the reactions shown in Scheme 1.

Several studies regarding viologens have revealed their vulnerability and instability in the presence of oxygen and other radical scavengers. This capacity has been successfully exploited to develop strategies of paramount importance to stabilize the photogenerated radicals. Due to their inherent porosity, MOFs can also be loaded with photoactive materials such as the viologen cations that undergo some electron transfer upon irradiation in a similar way to molecules that are known to change color due to a *cis*–*trans* isomerization process. The success of this method depends entirely on the size of the pores within the MOFs [34].

Devices using water-soluble, chromogenic thiazolo(5,4-d) thiazole (TTz) dyes were developed by a research group headed by Michael Walter [35]. They synthesized viologens from TTz and these were later studied in PVA/borax hydrogel devices from which the ease-to-assemble and high oxidative stability in the air were highlighted. These multifunctional chromogenic devices (CGDs) were efficiently reversible and stable, with valuable fast-switching steps. They also exhibited stable multichromic properties.

The MOF material with the methyl viologen (MV) [Zn_3_(IPA)_4_ MV] (H_2_IPA = isophthalic acid, MV⋅2NO_3_ = 1,1′-dimethyl-1,1′-diium dinitrate), constructed in coordination with Zn and isophthalic acid, was developed by Liu et al. with the purpose of being used in inkless and erasable printing. This compound showed fast-responsive reversible photochromism under UV-Vis radiation due to the electron transfer between the host skeleton and guest MV^2+^ [36].

A particular and interesting photoswitching mechanism that resulted in the modulation of the MOF’s properties without changing its structure was reported. In this context, UV-triggered inter-linker electron transfer was favored by the pseudo-rotaxane assembly of an Eu-containing MOF. Viologen radicals resulted from this process, which conferred a blue color to the material and quenched the fluorescence of the Eu(III) cations [37].

The study of the redox processes in electrochromic materials is fascinating. These materials can reversibly change their color due to an electrochemical reaction and are an essential component in all kinds of ECDs. To develop and produce tailored ECDs, it is important to understand the mechanisms of the electron transfer reactions in such materials and to identify the species responsible for the coloration.

Considerable research has been recently conducted to study the integration and synchronization of controlled molecular motions, as described by the research team of Giuseppone [38]. One of their proposals implies classifying the molecular structures according to their hierarchical dynamics, potential functions, and applications at micro- and macro-scopic scales. Typically, molecular switches are defined as chemical species possessing at least two thermodynamically (meta)stable states and with the characteristic of reversible interconversion to external stimuli. Such switches are typically activated by various chemical, molecular electrochemical, or photochemical stimuli. One of the most relevant structural factors is that their isomers should be stable enough to avoid spontaneous thermal relaxation [38].

Recently, several investigations have focused on the production of redox-active viologen units at the heart of a dumbbell-shaped molecular pump. Developments have also been directed toward the design of novel pump systems by attracting and then repelling the rings during redox cycling. This mechanism enacts flashing energy ratchet cycles with a minimalistic design to compartmentalize highly charged rings in a high-energy state. For instance, a valuable synthetic strategy has been established to succeed in this biased operational principle. It can be envisaged as an element of a molecular pump that would concentrate highly directed macrocycles. Several authors have worked on pumps and motors inherent in biological systems [39,40,41]. A clear example showing an oxidative transformation is indicated by the chemical or electrochemical oxidation of tetrathiafulvene (TTF). Since the TTF unit is more π-electron rich than the 1,5-dioxynaphthalene (DNP) one, the electron-poor cyclobis(paraquat-*p*-phenylene) (CBPQT) ring preferentially encircles the TTF unit rather than the DNP one in the starting co-conformation.

**Figure 1:**
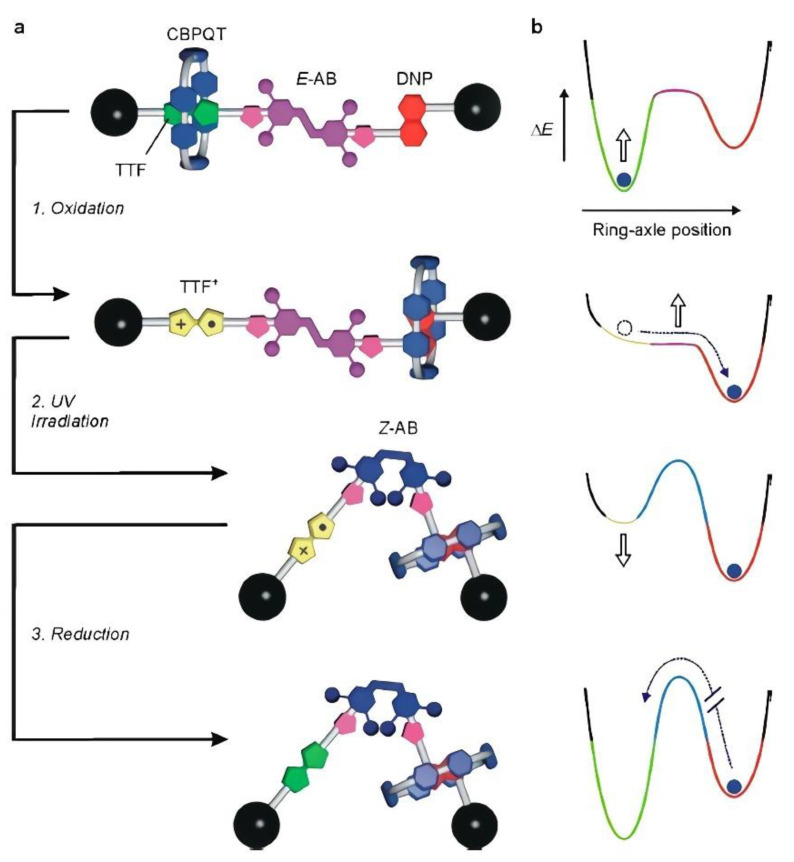
Schematic representation of the redox and photochemically triggered switching processes in rotaxane 1^4+^. (**a**) Initially, the CBPQT ring encircles the TTF site on account of stronger π-electron donor–acceptor interactions than with the DNP site. Oxidation of the TTF site while the azobenzene unit (AB) is in the E configuration (process 1) is followed by the fast translation of the ring to the DNP site by Brownian motion. Irradiation of the oxidized rotaxane at 365 nm causes the isomerization of the azobenzene unit to the bulkier *Z* configuration (process 2) without affecting the position of the ring along the axle. Upon regeneration of the TTF primary station by reduction (process 3), a driving force for the return of CBPQT on the TTF site is created; however, because of the presence of the *Z*-AB stopper, the ring remains blocked in a nonequilibrium state. Therefore, as shown by the simplified potential energy curves (**b**), the macrocycle is moved energetically uphill by an energy ratchet mechanism. Reproduced with the permission of the American Chemical Society from [42]. Copyright 2019, American Chemical Society.

Consequently, the potential energy determined by the donor–acceptor interaction with the CBPQT ring is canceled, and a driving force for the motion of the ring toward the DNP unit is created, as illustrated in Figure 1 [42]. Without equivocation, this finding can be revealed by the *E*→*Z* isomerization of the azobenzene unit affording a large geometrical change capable of substantially affecting the energy barrier for the shuttling of the macrocycle along the axle. Furthermore, the significant structural feature of the [2] rotaxane (*E*)-1^4+^, a photoactive 3,5,3′,5′-tetramethylazobenzene moiety (AB), has the ability to be switched between the *E* and *Z* configurations due to the effect of light irradiation; this is located between the TTF and DNP units, as shown in Figure 1.

Lately, the efficiency of supramolecular gels used in catalytic processes has been corroborated in an exhaustive study presented by Professor D’Anna and her collaborators [43]. In another instance, light responsive components might include the biological systems, as established by Chen Wang, who performs a unique review about DNA-based hydrogels with transient behaviors by responding to self-regulating chemistry. In this review, the authors discuss the response of DNA-based hydrogels to various stimuli [44].

There are several experimental investigations as well as mathematical modeling that have brought many new findings and suggestions for further research. The incorporation of the chromophores via covalent bonds into perfectly ordered molecular alignment helps to improve the photoswitchable control, including for metal–organic systems.

## 3. Photoswitchable Control of Supramolecular Organic Systems

Considering the superiority of photoresponsive supramolecular switches, we will summarize recent progress to provide a comprehensive overview of these host–guest systems with the peculiarity of reversibly shifted and present different structural states by light stimuli. Such photoswitchable systems have been applied both in tissue engineering and drug delivery and to produce smart materials. In addition, we approach a range of applications of self-assembly materials such as metal–organic cages (MOCs), metal–organic frameworks (MOFs), and hydrogen-bonded organic frameworks (HOFs) and their opportunity schemes as the next generation of materials that respond to external stimuli and that are part of the new technologies that inspire new research ideas.

### 3.1. Metal–Organic Polyhedra (MOP)

Several research groups around the world have dedicated a large space in their agendas to synthesizing and establishing broad interrelated characterization routes on metal–organic polygons and polyhedra (MOPs), namely squares, cubes, tetrahedra, and hexahedra. A large number of studies revealed the possibility to achieve desired structures constructed from nodes of either single metal ions or metal carboxylate clusters connected by organic links [45]. In this sense, the construction of self-organizing systems concerns the reticular chemistry that links molecular building units through strong bonds forming predefined structures. The research on reticular synthesis mainly focuses on 2D or 3D extended frameworks; according to these properties, each molecular unit in reticulation is built with the functionality demanded to form specific linkages chemically and geometrically to shape the framework [46,47].

The great structural design of the MOP and their ability to form several topological structures has been exhaustively covered by Lee et al.; they extensively explain common designs, such as regular convex polyhedra or “Platonic solids”, and which consequently have one kind of vertex, one kind of edge, and one kind of face. There are five different regular polyhedra with transitivity values of (1, 1, 1): the tetrahedron (tet), cube (cub), octahedron (oct), dodecahedron (dod), and icosahedron (ico). The second essential polyhedra are “quasiregular” polyhedra, which have one kind of vertex and edge but two kinds of face, and therefore a transitivity of (1, 1, 2), such as the cuboctahedron (cuo) and icosidodecahedron (ido). In addition, there are two more edge-transitive polyhedra with a transitivity (2, 1, 1), which present one kind of edge and one kind of face, such as the triacontahedron (trc) and rhombic dodecahedron (rdo) [48]. Only fourteen types of polyhedra have been discovered in MOP assembly compared to innumerable topologies of MOFs known. Moreover, the captivating symmetric topologies and the discrete structural features limit the applications of MOPs, unlike MOFs.

Specifically speaking, microporous metal–organic polyhedra are similar to supramolecular cages in such a way that they can be accurately defined as a subset of them. In this regard, pioneering works on the syntheses of tetranuclear magnesium-based tetrahedron and palladium are systems broadly representative of the class of coordination cages [49]. Following the same line of research, the work carried out by Jiang et al. is remarkable [50]. They emphasize that the confinement strategy of a PMOP in nano-scaled spaces of MS endows photoresponsive MOPs with real photoresponsiveness and, consequently, they have control over their photoresponsive efficiency.

There are characteristics linking chemical structures that have resulted in the recent development of orthogonal surface chemistry and the assembly of more stable cages, and for the same reason, it has been established that the MOPs are a subclass of coordination cages [51,52].

Cage-like molecules, built by the coordination of organic links and metal ions, are opening new horizons in material science due to their design flexibility. Metal–organic cages (MOCs) are systems with a high degree of symmetry, which is promising for several applications such as separation, adsorption, catalysis, molecular recognition, chirality sensing, and reactive species stabilization [53,54,55]. MOCs are often produced using self-assembly mechanisms, in which components undergo a reversible process that leads to thermodynamic equilibrium. Chemists have largely avoided some of the complications of MOC formation by concentrating on rigid and symmetrical building blocks, metals with well-defined geometries, and the design principles that result from those choices. However, there has been a trend to develop more sophisticated and functional MOCs by self-sorting diverse ligands into heteroleptic cages, unsymmetrical ligands into asymmetrical MOCs, or ligands with secondary functionalities into functional materials. The extremely large availability of MOC precursors allows researchers to tailor them for specific properties [56,57]. Moreover, their discrete, rather than extended, structures with well-defined shapes, specific cavities, nano-scale sizes, and symmetrical geometries allow them to be used as self-sufficient materials in a wide range of applications but also to tune soft materials used in medicine, robotics, and battery research [58].

Recently, MOCs have been studied as photoresponsive materials whose properties depend on light radiation. The ability to properly control the structure of MOCs by external stimuli opens a wide research line on applications such as product inhibition during catalysis, drug release, and photocatalysis by controlling the molecule architecture and, hence, their uptake, delivery, and release of cargo features [55]. In Table 1, we report data from several articles, and this includes the coordinating metals, organic ligands, cage structure, reaction times, reusability, geometry, and applications. Palladium and copper are common resources for building MOCs as they have strong coordination capabilities with anionic moieties and their multiple ionic states, resulting in up to ten coordinated stable states [59,60]. Most of the revised articles used 365 nm wavelength UV light for promoting forward reactions, while the backward reaction can be stimulated using green with blue or another type of light [59,60,61,62,63]. The reaction times can vary from a few minutes to several hours, making this characteristic a critical variable for developing trustworthy materials.

Generally, these stimuli-responsive materials present metastable states because the external stimuli are present; upon removal, the materials return to their initial state [60]. In their work, Yuwei Gu et al. [70] state that, in many systems, the operational conditions are defined by the material compositions and topology, or their response may be limited to a single network property. They studied cooperative self-assembly as a principle for photoswitchable topologies in smart materials containing MOCs. The materials’ design included the synthesis of a poly(ethylene glycol)-based polymer ligand including two bis-pyridyl diethylene (DTE) groups and Pd^2+^. This system reversed the photo switch between large Pd_24_L_48_ rhombicuboctahedron rings and small Pd_3_L_6_ rings during green and ultraviolet light irradiation. The rheological studies showed that the material displayed an elastic behavior in frequency sweep tests along with the entire frequency range with a storage modulus (G′) of about 8.3 kPa at 10 rad/s when no stimuli were applied. After UV irradiation for 5 h at 60 °C, a dark-blue material was produced with a practically double G′. This change was attributed to the presence of greater topological defects due to the formation of larger MOC structures. A subsequent green light irradiation for 5 h at 60 °C decreased the stiffness to that obtained with prior irradiation steps (G′ = 7.8 kPa at 10 rad/s). Finally, the photoswitching topology led to tunable network dynamics capable of producing self-healable and non-healable gels.

**Figure 2:**
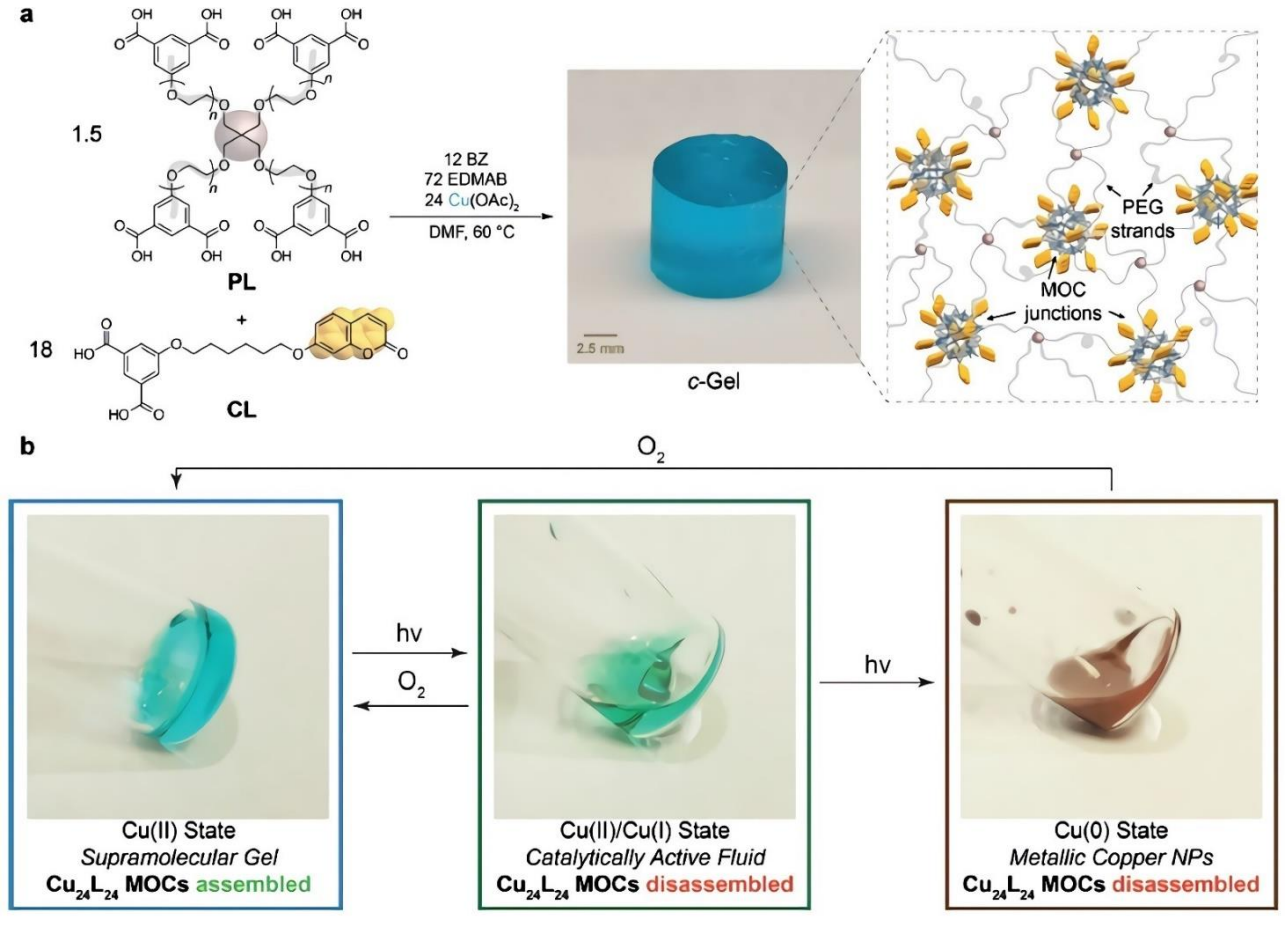
Design of Cu_24_L_24_-based polyMOCs featuring coumarin-functionalized junctions. (**a**) *m*-BDC-functionalized PEG star polymer PL and coumarin-functionalized *m*-BDC (CL) are combined with Cu(OAc)_2_, dimethylformamide (DMF, solvent), benzophenone (BZ, photosensitizer), and ethyl-4-(dimethylamino)benzoate (EDMAB, H-atom donor). Annealing provides coumarin-functionalized polyMOC *c*-Gel, composed of Cu_24_L_24_ junctions decorated with coumarins and polymer strands from CL and PL, respectively. (**b**) Photoreduction or air oxidation enables reversible interconversion of *c*-Gel between Cu^II^, Cu^I^, and Cu^0^ functional states with corresponding gel–sol transitions and differences in catalytic activity. Reproduced with the permission of Wiley-VCH Verlag GmbH & Co. KGaA, Weinheim from [60]. Copyright 2019, John Wiley & Sons.

Besides self-healing applications, several researchers have been investigating MOCs for catalysis purposes. Oldenhuis and co-workers reported the synthesis of a Cu_24_L_24_ metal–organic cage where coumarin-based compounds were used as ligands [60]. The resulting cuboctahedral cage was crosslinked by a four-arm star PEG polymer to form a gel system. When subjected to UV light in the presence of a photosensitizer and a hydrogen donor, the system is reversibly photo-reduced and switches between Cu(II), Cu(I), and Cu(0) ions. Such a mechanism represents a tool to control the gelation process by the reversible disassembly of the supramolecular cage. The stable redox states of the system are observed as a Cu(II) viscoelastic solid and two fluid phases named Cu(II)/Cu(I) state and Cu(0) state. The Cu(II)/Cu(I) state was catalytically active. As proof of the Cu(II)/Cu(I) functional state, they catalyzed the formation of a covalent polymer network via azide-alkyne cycloaddition (CuAAC). On the other hand, the Cu(0) state resulted in a suspension of Cu nanoparticles with no catalytic activity. Interestingly, both fluid states were reversible to the Cu(II) state, as shown in Figure 2. They also reported four cycles of use without adding new reagents, corresponding to twelve different state transformations in a single sample.

Other research focused on catalysis is the one performed by Zhang and co-workers who developed a cage-like structure using the melamine-based ligand H_6_TDPAT (2,4,6-tris(3,5-dicarboxylphenylamino)-1,3,5-triazine) and Cu(II) salt to form the coordination polymer Cu*-*TDPAT [65]. The phenomenon behind this proposal is the photoinduced electron transfer from the ligand to the Cu(II) node of the polymer leading to an excited state. Then, triggered by removing the stimuli, there is an electron back transfer, and the ground state is reached. The ligand-to-metal charge transfer enables the Cu(II)/Cu(I) switching in response to light. The reported application of such a supramolecular switch at the ground state Cu(II) is the CO_2_ cycloaddition to allylamine. In contrast, under photoirradiation, Cu(II) is switched in situ to Cu(I), which undergoes a reduction reaction when in contact with Togni’s reagent II, triggering the radical trifluoromethylation process. This achievement represents an efficient catalytic system that merges CO_2_/CS_2_ reagents and CF_3_ radical in a single scaffold with potential use in the environmental and pharmaceutical industries.

MOCs as photosensitive materials for photoswitching supramolecular applications have plenty of room for research due to the vast availability of metal and ligand precursors. However, almost all the cases included in this revision are basic proof-of-concept investigations. In order to assess the practicality of using these materials in the industry, a broader consideration of their stability, toxicity, and potential environmental harm, as well as their cost, will be necessary. Considering the scale-up of separation processes and the recovery of the cages in larger-scale separation scenarios will be critical to extending the application of MOCs to real industrial processes, building on the approaches that have already been developed at a laboratory scale.

### 3.2. Metal–Organic Framework (MOF)

MOFs are inorganic–organic hybrid materials with varied structural geometries and, consequently, due to their enormous surface area and functionality, these materials have highly targeted applications.

MOFs, also known as porous coordination polymers (PCPs), are assembled from metal ions or clusters and organic linkers via metal−ligand coordination bonds and have captivated significant scientific interest on account of their high crystallinity, exceptional porosity and tunable pore size, high modularity, and diverse functionality. However, to date, the potential of chiral MOF (CMOF) materials for specific applications include chiral recognition, separation technologies, and catalysis [71]. Regarding the above, the definition that has been best carried out for MOFs is the one that Barton and collaborators very accurately expose. They correctly establish the topologic descriptors to enhance crystal structures of MOFs and 3D coordination polymers [72].

According to Ma et al., MOFs and covalent organic frameworks (COFs) are two classes of crystalline polymeric and porous materials. On the one hand, the MOFs are assembled by metal ions/clusters and organic linkers; on the other hand, the COFs are constructed by pure organic building blocks [73]. Using the technologies of design MOFs and COFs, they perform a great job of reviewing the anchoring aggregation-induced emission (AIE) and AIE luminogens (AIEgens) in those structures.

AIEgens and the corresponding supramolecular materials expand the detailed information beyond the fundamental insights and into the self-assembly of nonplanar molecules and the frontiers of supramolecular chemistry. AIEgens are likely to undergo photoexcitation due to their restricted intramolecular movements in the presence of neighboring molecules [74]. The design and synthesis specific to AIEgens with photocontrollable emissions covering violet, blue, green, yellow, orange, red, deep red, and NIR regions have been associated with the HOMO-LUMO energy level by donor (D)–acceptor (A), exemplified by the blocks formed by phenylamine (TPA)–thiophene [75]. Cytotoxicity studies in HeLa cells were evaluated using pyridinium-substituted tetraphenylethylene salts-based compounds (identified as *TPEPy*-I and *TPEPy*-PF6 by the authors), whose efficacy was stipulated by Lingyun Wang and Wei Chen. The authors made it clear that significant cytotoxicity of AIGens occurs when they are excited by microwave (MW) towards HeLa cells, even at low concentrations. Under these assertions, the average HeLa cell viabilities turned out to be 58.4% vs. 62.5%, 31.1% vs. 36.7%, and 9.3% vs. 14.2% at 2.5, 5, and 10 μM of the iodine compound *TPEPy*-I and *TPEPy*-PF6 with hexafluorophosphate in its structure, respectively, under 10 W (2450 MHz) of MW irradiation for 1.5 min [76,77].

The synthetic route to obtain the products *TPEPy*-I and *TPEPy*-PF6 has been reported in detail, where it predominantly involves the Suzuki reaction using 5-formyl-2-thiopheneboronic acid and 4,4′,4″-(2-(4 bromophenyl)ethene-1,1,2-triyl)tris(methoxybenzene) through acceptor structural intermediates such as tetraphenylethene, thiophene, and aldehyde, and thus obtains a robust starting compound harboring donor-π-acceptor units [78,79].

Undoubtedly, the combination of materials, organic and inorganic, is the key element in COFs, and the emphasis devoted by the scientific community to making optimal topologies of two dimensions (2D) is greatly determined by the connectivity and symmetries of the building blocks involved. Therefore, the morphology and symmetries of building blocks of a 2D COF have relevant implications on its topologies and structural networks. Going into detail, Peng et al. show a procedure for preparing flexible conformation 2D COFs that can provide additional degrees of freedom for their topologies. Specifically, the products were synthetized from tetrakis(4-aminophenyl)ethene [80] with 2,3-dihydroxyterephthalaldehyde (2,3-DHTA) and with 2,3-dimethoxyterephthalaldehyde (2,3-DMTA) to obtain *TPE-COF*-OH and *TPE-COF*-OMe, respectively, with different topologies and porosities. This was achieved through the activation or passivation of intramolecular hydrogen bonding, which requires looking deeper into the conformation of molecular linkages in the networks, leading to the topology regulation of COF networks [81].

In various aspects of the nature of energy-emitting centers, electron transfer is inherent in π-conjugated systems; that is, the combination of interactions of chemical structures where some are donors and others are acceptors facilitates intramolecular charge transfers (ICT), which systematically result in low electronic band gaps, broad absorption, and long emission wavelengths [76].

The use of AIEgens to structure MOFs and COFs through non-covalent interactions and well-established interactions has been described, and not in an unpredictable structural way as presented by the AIEgens [73]. The well-established strategies for the design of materials such as connecting AIEgens based on reticular chemistry can be understood by the thermodynamics and the kinetics of the reaction via altering the reaction conditions, and crystalline AIEgen-MOFs or AIEgen-COFs with distinct topologies can even be made using the same organic building units and metal sources.

Increasing productivity in material synthesis and the characterization for evaluating the photoisomerization and physical behaviors of an imine-linked photoresponsive COF have given way to significantly planning the reaction between dithienylethene-dialdehyde and 5,10,15,20-tetra(4-aminophenyl)-porphyrin (H_2_TAPP) [82].

Developing stimulus-responsive (e.g., an external light radiation) chiral co-assemblies facilitates the fabrication of smart chiroptical systems with exceptionally high quality by changing the intrinsic properties [83]. Implications on a material structural can be deepened for the generation of circularly polarized luminescence (CPL) based on a binary system composed of nonchiral, small molecular fluorescent dyes, and chiral nanofibrillar templates. For instance, the morphologic design of complicated molecular fluorophores is practically excluded in this system because chirality is not an essential requirement for the fluorophores [84]. As far as the intrinsic structure of an active material is concerned, electrostatic attraction and charge transfer can combine multiple components into a cooperative assembly. Likewise, intermolecular, non-covalent interactions such as π−π stacking, hydrogen bonding, and halogen bonding, must be deeply investigated.

For instance, an investigation carried out by Alaasar culminated in the production of supramolecular polycatenars formed by an intermolecular halogen bond between two different types of azopyridine derivatives, terminal alkoxy chains, and an iodo-tetrafluoroarene ring as the halogen-donor [85]. This is an excellent way to form compounds that make use of halogen bonding for elaborating liquid crystals. On the basis that the halogen bonds can have a direct influence on the dynamic processes, the halogen bond donor acts as a dynamic superior catalyst to the hydrogen bond. Based on this, the dynamics catalysis by the reduction of the energy barrier associated with a dynamic process such as molecular rotations is favored [86].

Discovering new compounds and materials continues to be a time-consuming, trial-and-error process. A very well-detailed example is the one reported by Bashir et al. They evaluate the antioxidative properties of magnoflorine (MF), a quaternary aporphine alkaloid. This was achieved by synthesizing MF-loaded chitosan−collagen nanocapsules (*MF*-CCNc) for a drug delivery system in order to increase the efficacy of MF against oxidative stress and to further enhance its delivery to the brain. Surprisingly, the synthesized nanocapsules (*MF*-CCNc) showed good encapsulation efficiency around 76 ± 1%, which substantially decreased its toxicity [87].

A major focus will be on the researcher. Up to now, using several combinations of atoms to discover new self-assembly materials looking for a multifunctional metal–organic framework (MOF) by developing methods that extend beyond the conventional has been the center of several advanced molecular designs. This should be understood in accordance with the powerful process of assembled structures and their conversion into nanostructured materials. These functional materials can be also coupled with other related approaches building on molecular architectonics by combining nanotechnology-oriented strategies and supramolecular-chemistry such as the methodologies described by Ariga and Shrestha [88].

It is possible to perform chemisorption and physisorption of the electroactive O_2_ molecules in a representative 2D-layered MOF, namely, Co_3_(HTTP)_2_ ((HTTP = hexathiotriphenylene). It is to be considered that the performance is not only due to open-Co(II) sites but also because redox-active HTTP linkers take part in the adsorption of electroactive dioxygen by forming strong Co=O and S=O bonds [89]. The group of Jihan Kim has reported the photoresponsive compound azopyridine-attached Mg-IRMOF-74-III for post-combustion carbon capture. They detailed the structure by DFT and Monte Carlo simulations, revealing a significant adsorption ability and an optimized desorption capacity [90].

From the perspective of synthetic planning, distinct ambitious and progressive features such as the switchability of chiral p-stacked conjugated molecular components for the MOFs are of great benefit [91]. The variety of topics and their fascinating positive impact are really invigorated by the micro-supercapacitors made of carbide-derived carbons and 2D materials including graphene, MXene, metal oxides, and conductive MOFs, which are among the most exploratory flexible and effective integrated energy storage devices. In this regard, a studied pathway to achieve this is manipulating the electrolyte for example by using thermally responsive polymer gels to control the ion transport between the electrodes, which can eventually cause the on and off switching of the device [92] and aligned carbon nanotube-poly (2-hydroxyethyl methacrylate) (*CNT*-PHEMA) [93].

MXenes is a 2D nanomaterial represented as M_n+1_X_n_Tx (n = 1–3) and fabricated by etching the A layer of MAX phase, where M is an early transition metal element (e.g., Sc, Ti, Zr, V, Nb, Ta, or Mo), X is carbon and/or nitrogen, A is a main group element (IIIA or IVA), Tx usually refers to some surface termination groups introduced after HF etching, such as -OH, -O, and/or -F [94,95]. Extensive studies in highly relevant applications for electromagnetic interference (EMI) shielding implementation have been developed such as MXene/Graphene nanoplatelets (GNPs) composites among others; namely, *MXene*-polymer composites, based on polypyrrole (PPy), polyvinyl alcohol (PVA), and sodium alginate (SA) [95].

A novel photoresponsive organic–inorganic hybrid ferroelectric material, whose composition is (Me_2_NH_2_)[NaFe(CN)_5_(NO)], is formed by assembling an inorganic photochromic nitroprusside anion, as the framework building block, and a polar organic cation Me_2_NH_2_^+^, as the dipole-moment carrier, into the crystal lattice. It is notorious that the ferroelectricity property is observed by the synergetic ordering of Me_2_NH_2_^+^ below 408 K [96]. In the recent literature, there are examples of the application of MOFs in sensors; in this regard, in biomedical devices, for example in chemosensory, the interactions of affinity and selectivity should obviously not have a practical limitation for an accurate diagnosis. Rapidly expanding discoveries such as studying the switching of azobenzene units within the strictly crystalline, highly porous environment of MOFs have significantly boosted scientific knowledge and research regarding the study of activation barriers for conformational changes generally applicable for any photosensitive molecules [97,98,99,100,101].

In summary, the setting up an MOF must be as simple as setting up a conventional structure. However, as we already know, intramolecular interactions are essential to obtain specific geometries in countless architectural variants and their impact on perfectly designed applications.

### 3.3. Hydrogen-Bonded Organic Framework (HOF)

It is interesting to note that in porous framework structures such as MOFs and COFs the specific geometry and interconnected voids allow for the adsorption of foreign molecules to its molecular structure. The same thing happens in hydrogen-bonded organic frameworks (HOFs); the discrete guest molecules can include organic solvents and aromatic compounds, and other molecules either in liquid or solid form with interaction properties are well tailored. The aim of the topology design is to elaborate the covalent bond formation and to guide the polymer backbone growth, which can be regulated externally with light irradiation to ensure a clear direction of each covalent bond. In this context, the monomers are required to have relatively rigid backbones in which the reactive sites are distributed in a distinct conventional geometry [102].

**Figure 3:**
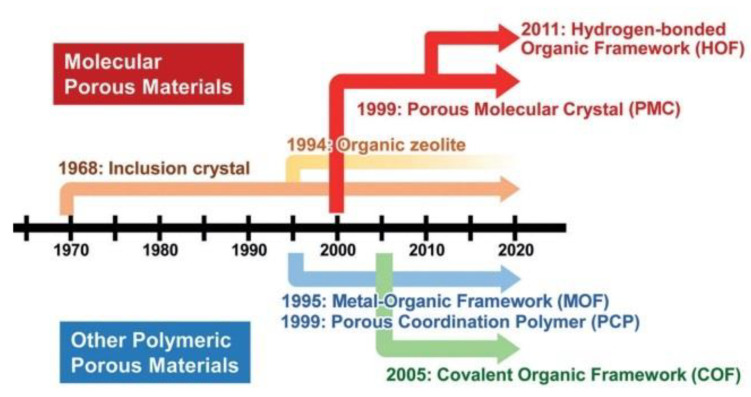
Representative timeline for the development of types of porous molecular materials and related materials. Reproduced with the permission of Wiley-VCH Verlag GmbH & Co. KGaA, Weinheim from [103]. Copyright 2019, John Wiley & Sons.

A timeline that indicates how the types of porous molecular materials and related materials have evolved was detailed by Hisaki and co-workers [103], as shown in Figure 3.

Several design criteria have been established to enhance PMCs, which involve H-bonding to form a framework with permanent porosity, and these materials were termed hydrogen-bonded organic frameworks (HOFs) by Chen [104]. The perspective in this area is incorporating geometrically rigid metal complexes into HOFs to maintain permanent pores and expand functional sites. Progress in the knowledge about HOFs, of course, is dependent on the development of structural complexes through non-covalent interactions between molecules with specific shapes [105].

It should be noted that hydrogen bonds in HOFs are relatively weak, and this impacts structural stability. Considering this, recently, Ke and co-workers prepared some HOF complexes with aniline, o-, m-, p-toluidine, toluene, and *N*-vinyl-2-pyrrolidone. The authors explain in understandable terms that the strong HOF−substrate interactions, including hydrogen bonding and N-H···π interactions, direct the assembly of guests as cyclic tetramers within the 1D voids of HOFs. The conclusions were obtained with the help of single-crystal X-ray diffraction (SCXRD) analysis [106]. Additionally, it is noteworthy that the interactions of cyclic tetramers with the HOFs are further stabilized through the guest−guest interactions, such as CH···π and van der Waals interactions.

Luminescence is a general term that describes any nonthermal processes in which energy is emitted from a material at a different wavelength from that at which it is absorbed. An example exists in a paper that exposes a HOF synthesized from *N*,*N*′-bis(5- isophthalic acid)naphthalimide (H_4_L). When immersed in an aniline solution, the single crystal changed from light yellow to deep red, and the transformation from single crystal to single crystal occurred. Obviously, this material can be used as a sensor with specific properties. Consequently, because the aniline forms a hydrogen bond with the framework, the twist of the skeleton is restricted [107].

The supramolecular chemistry group of Nicholas White prepared a family of robust hydrogen-bonded network materials using the amidinium–carboxylate interaction. They were able to vary the structure of both the amidinium or carboxylate tecton and introduce additional functionality while still retaining predictive power in terms of the framework structure. According to them, their pioneering work has the characteristics of representing an enormous potential for synthesizing hydrogen-bonded materials, with potential applications for challenges in materials chemistry [108,109]. Regarding tectons, factors such as its structure, the topology, and the geometry will affect the responsiveness and mechanisms for understanding the implicit transformation of any process using HOFs [110]. It is feasible to point out that tectons are molecules with specific interactions that induce the assembly of aggregates perfectly designed to establish specific geometries. There is remarkable evidence that tectons can participate in extensive networks of hydrogen bonds with a special group of molecular frameworks [110].

An ionic HOF, termed *HOF*-ZJU-102, which combines the bond-connectivity type of both MOFs and HOFs, has been reported by Liu et al. [111]. It is possible to construct from the cationic metal–organic complexes [Cu_2_(adenine)_4_(H_2_O)_2_]^4+^ and inorganic GeF_6_^2−^ anions by strong ionic H-bonds. Structures on the metal–organic complexes act as H-bond donors [D-H]^y+^ and inorganic anions act as H-bond acceptors [A]^x−^. In this context, previous studies have pointed out a HOF assembled from [Cu_2_(adenine)_4_] using SiF_6_^2−^ anions [112]. The resulting HOF [Cu_2_(adenine)_4_(H_2_O)_2_]·2SiF_6_, termed HOF-21, has a significantly different structure from [Cu_2_(adenine)_4_(TiF_6_)_2_] (*MPM-1-*TIFSIX) compound previously reported by Nugent et al. [113], suggesting the occupancy of water molecules on the copper sites.

Up to this point, there is evidence of overwhelming information indicating that the compression of the formation of an HOF and its stability depends on its rigidity and the strength of the backbone structure and the number of hydrogen-bonding sites [114,115].

Owing to its major technological implications, the fundamental aspects associated with MOFs and HOFs have been scrutinized by Zuo and co-workers, who designed a MOF-template constructed from two coordinated Cu^I^ ions and tetrathiafulvalenetetrabenzoate (TTFTB) using the solvothermal method. Starting from the oxidation of material Cu^I^TTFTB, they obtained the intermediate MOF (Cu^II^TTFTB) and hydrated to the HOF product (*TTFTB*-HOF) by single-crystal-to-single-crystal (SC-SC) transformation. It should be noted that MOF to HOF transformation slightly increased the pore size and significantly enhanced the electron conductivity by about threefold to 2.9 × 10^−5^ S m^−1^ [116].

The most common area of HOFs has to do with predictable H-bonding. There are several advantages associated with various isostructural hexagonal networks (HexNets) of C3-symmetric carboxylic acids with p-conjugated cores. A key factor is the mosaic of materials has been formed using 1,2-diphenylethyne (tolane) and 4,7-diphenylbenzo-2,1,3-thiadiazole, which defines the relationship of carboxy-phenyl and -biphenyl arms [117].

Numerous fabrication processes have been developed in order to achieve the production of photochromic semiconductive HOFs of fundamental importance in materials science and technology. In this visualization, it is possible to use viologen units to prepare photochromic semiconduction with photoelectric response through a careful design of hydrogen-bonding networks. A significant contribution was made to obtain [H_2_(bpyb)](H_2_PO_4_)_2_·2H_2_O (bpyb = 1,4-bis(tetrapyridyl)benzene) using the polycyclic viologen cation [H_2_(bpyb)]. The HOF showed an absorption band of 200–1700 nm after xenon lamp irradiation [118].

More innovation and development are expected in the category of simple peptide building blocks with the intrinsic ability to self-assemble into ordered nanostructures. In this context, there are many advantages of the peptide self-assemblies that endow them with very particular and diverse properties, namely quantum dots and semiconductive superstructures [119].

## 4. Azobenzene and Diethienyl Bearing Photoswitchable Catalysis

In the sphere of photoresponsive materials, the advent of nanostructured materials is considered decisive, especially in photochromatic technology, as *E* to *Z* transformation allows modifying the properties’ photopolymerization. Consequently, the materials have controlled optimal applications with structural designs with high specificity. In this chapter, we focus on azo and dithienylethene systems possessing an appreciable impact on the stereoselectivity of a reaction in response to light stimuli, including the changes in their electronic or steric properties to achieve the desired results in any catalytic reaction.

### 4.1. Azobenzene Photoswitchable Catalysts

Azobenzene is probably the most widely studied photoresponsive group to develop photoswitchable catalysis involving a photochromic moiety incorporated into the catalyst. The importance of azobenzene in catalysts is due to its low cost, ready availability, versatility, and facile development into several structures. On the other hand, light has many advantages over other external triggers, such as its non-invasive nature, easy handling, and its ability to exert a high level of control by tuning the wavelength or intensity of the light source [120]. Under visible light irradiation, the azobenzene molecule mainly exists in the form of trans-conformation. Under UV irradiation, trans-Azo-derivatives can be transformed into *cis*-Azo-derivatives [121]. Upon irradiation, the *N*=*N* bond undergoes a cis–trans isomerization, which reverts to the thermodynamically more stable isomer in the dark. This reverse isomerization can also be triggered by irradiation with visible light. Therefore, a new enhancement of the isomerization rate can be reached by simply modulating the so-called *E*-azobenzene to the *Z*-azobenzene systems.

Research in azobenzene photoswitchable catalysts is open to many possibilities for developing systems with precise control chemical reactions toward chemo-, regio-, and stereo-selectivity. However, in-depth research and comparative performances for photoswitchable catalysts based on azobenzene are still in their early stages [122,123,124,125,126,127]. In this regard, azobenzene single molecules, molecular assemblies, and molecularly printed azobenzene polymers and organometallics are developed as photoswitchable systems. In addition, azobenzene systems can also be immobilized onto the surface of nanoparticles to produce photoswitchable heterogeneous catalysts that can cause changes in reaction rates. In catalytic applications, the reaction rate can be photocontrolled by changing the diffusion kinetics of reactants, activators, or inhibitors to the catalyst surface, the aggregation state of catalysts, and the properties of active surface site catalysts [124]. The synthesizing of some azobenzenic systems with photoswitchable characteristics is reported in Table 2.

#### 4.1.1. Single Molecules

The reversible steric shielding of the catalyst’s active site by a suitable blocking group, which can be moved away from the reactive center using a photochromic linker, motivated Hecht and collaborators to develop the first photoswitchable catalysts based on conformationally locked piperidine bases [125]. The photocontrol of catalytic processes relies on geometrical changes of the catalyst’s backbone that alter its interaction with the substrate. This approach allows the photoswitchable catalyst to adopt two states (i.e., ON-state and OFF-state), whose reactivity is controlled by reversible steric shielding when irradiated using light. Extension of the photoswitchable concept with better ON/OFF ratios has been developed, focusing intrinsically on more reactive catalysts and more responsive materials to react with light. For instance, a photoswitchable catalyst was developed in the Morita–Baylis–Hillman (MBH) reaction [126] using a bifunctional molecule prepared from azobenzene-tethered bis-(trityl alcohol) with a function of cooperative acid catalysis. The differently arranged trityl alcohol units change their joint function to reflect the positional relationships. Light stimuli can control the activity as a cooperative acid. The switching is based on the photo-induced reversible molecular motion related to the photoisomerization of azobenzene. This way, the two trityl alcohol groups are arranged differently on the resulting isomeric catalyst, leading to a switching catalytic. The ON-state is related to the *cis* configuration due to a cooperative function among the hydroxyl moieties. In another study [127], the photoswitchable activity for azobenzene-3,3′-dicarboxylic system acid was observed in its acid–conjugate base form as a glycosidase mimic with the p*K*_a_ modulation of two carboxyl groups through photoisomerization. After irradiation at 366 nm and 0 °C, the azobenzene system revealed the two distinct p*K*_a_ values of 4.7 and 6.5. According to the authors [126], the deprotonation of the two carboxylic groups took place stepwise in the *E*-azobenzene. At the same time, the azobenzene system exists in an acid–conjugate base form (–COOH and –COO^−^). The data suggested that the *Z*-azobenzene did not follow the kinetics pattern. It can be considered that this azobenzene system is the first development of a photo-controlled glycosidase activity with an enzyme mimic.

Azobenzene-based artificial pyridoxal phosphate was prepared from pyridoxal-5-phosphate-azobenzene-imidazole triad as a photoswitchable racemase mimic [138]. The azobenzene triad can transform L-amino acids to their D-isomers and control the racemization reaction by adjusting the distance between the basic residue and the active site. In this study, it was demonstrated that the *cis* isomer effectively deprotonates and racemizes L-alanine, but the *trans* isomers are inactive for this transformation. A site-selectivity of catalytic acetylations of partially protected sugars was studied using a photoswitchable catalyst based on an azobenzene–peptide backbone [141] with π-methyl-L-histidine as the active site. The control of site-selectivity in catalyzed acetylation reactions validated this new catalyst design concept as it demonstrated the regioselective acetylation of the natural product quercetin.

One of the earliest examples of photoswitchable catalysis based on a conformational change induced by *trans*–*cis* isomerization of azobenzene was reported in the 1980s using β-cyclodextrin systems to conjugate azobenzene systems that act as capping units. According to Ueno and colleagues [142], the hydrolysis of p-nitrophenyl acetate catalyzed by β-cyclodextrin can be photomodulated through *trans*–*cis* photoisomerization of the potassium 4-(pheny1azo)benzoate-capped β-cyclodextrin. This system is based on allowing the cavity of β-cyclodextrin to receive the substrate-binding site in the *cis*-isomer. At the same time, this is not possible in the case of the *trans*-isomer because the cavity of β-cyclodextrin includes an azobenzene moiety. Finally, azo-crown, ether-based photoswitching systems were reported to enhance their catalytic activity using azobenzene. In a recent study, Kondo and colleagues [143] reported the preparation of an azobenzene binaphthyl crown ether that can control the reactivity of the enantioselective alkylation of a glycine Schiff base with benzyl bromide with/without UV irradiation. The azobenzene system is a photoswitchable chiral phase transfer catalyst induced by *E/Z* photoisomerization of azobenzene.

#### 4.1.2. Molecular Assemblies

Some azobenzene molecules have no catalytic properties, therefore self-assembled structures have been proposed as photoswitchable heterogeneous catalysts. This way, light-switchable catalysts with active sites on the surface of nanoparticles have attracted much attention due to the high density of active sites in the particle. The catalytic activity of peptide-based systems that can be reversibly controlled by light has been used to prepare azobenzene-based molecular assemblies with nanoparticles. For instance, the peptide *Azo-Gly-Phe-Gly*-*His* (*Azo*-GFGH peptide) shows a structural transition from antiparallel β-sheet to random coil under light irradiation, resulting in the assembly/disassembly of peptide fibrils [144]. Due to the proximity effect of histidine residues in supramolecular assembly and hydrophobic microenvironment, the assemblies have high catalytic activity for the hydrolysis of 4-nitrophenyl acetate.

In another study [141], the azob137enzene molecule terminated with a proline was immobilized on the surface of gold nanoparticles (AuNPs) via the Au-S bond. The catalyst was attached to the nanoparticle surface through ligand exchange via a specially designed linker. It comprised: (i) an azobenzene photoswitch that undergoes significant geometric restructuration, enabling the change in catalyst position in space; (ii) an aliphatic spacer, which mitigates the quenching of excited states of the chromophore by the gold core; (iii) a thiol group, which holds firmly the thus synthesized ligand (AzoPro) on the nanoparticle surface. Depending on the conformation of the photoswitch, the catalytic center is hidden or exposed to the reaction mixture, causing changes in the reaction rate. In this example, AuNPs were passivated with proline moieties and such surface immobilization was successful as the organocatalysts were effective. Finally, the rate of the chemical reaction can be reversibly regulated by light by appending the molecular catalyst on the nanoparticle surface via an organic photoswitch. Zhang and colleagues [140] recently reported the preparation of a light-switchable, cofactor-free, organic nanozyme by self-assembly of cyclodextrins-modified gold nanoparticle (AuNP@CDs) and azobenzene-modified peptide chain (Azo-GFGH) through host–guest interaction. Through light regulation, the catalytic activity of AuNP@*CDs-Azo*-GFGH under the alternate irradiation of UV and visible light forms a reversible cycle. According to the authors, the reversible regulation of catalytic activity of the nanozyme is achieved by changing the host–guest interaction of azobenzene and cyclodextrin. In contrast, the photoisomerization of the azobenzene molecule leads to the change in catalytic activity of nanozyme under UV/visible light irradiation.

#### 4.1.3. Other Systems

Catalysts based on organometallics are composed of two complexes in one molecule that confer a photoswitchable cooperative function. This way, azobenzene-containing ligands have a significant effect on light-induced ligand dissociation. For instance, an azobenzene–dinuclear Ru(II) complex was formed via the catalytic dimerization of aryl azides to produce azobenzene derivatives [137]. The complexes were tested as precatalysts for photo-controlled hydrogen generation by hydrolytic decomposition of ammonia-borane. It was observed that the *trans*–*cis* photoisomerization was inhibited upon coordination to the metal center in azopyridine-derived ligands, but it remained efficient in azobenzene-appended bipyridine and phosphine ligands. Another study showed that azobenzene-coupling cysteine dipeptides bound by Cu^2+^ undergo *trans*–*cis* photoisomerization [145]. The catalytic activity was approached as the copper complex in the *cis* form exhibited DNA cleavage activity. In contrast, the activity in the transform was negligible.

Photoswitchable catalysis mainly focuses on transforming small molecules using organocatalysts. This way, metal catalysts bearing azobenzene units for photo-controlled, ring-opening polymerization are rare. A series of photoactive Schiff base ligands bearing azobenzene moieties with Al^3+^ and Zn^2+^ complexes were reported by Kaler and colleagues [120] for selective ring-opening polymerization of ε-caprolactone and racemic-lactide (*rac*-LA). According to the authors, there were no differences in activity for *rac*-LA polymerization upon switching from ambient to UV light conditions. While, for ε-caprolactone (ε-CL), an apparent increase in catalytic activity was observed upon *trans*–*cis* isomerization under UV light.

Azobenzene derivatives (4,4′-diazene-1,2-diyldibenzoic acid, H_2_AzDC) have recently been used to functionalize MOFs and create a photoswitchable catalyst for the Knoevenagel condensation reaction [146]. The mechanism at the base of this photoswitchable behavior has been ascribed to the fact that H_2_AzDC located on the MOF pore wall isomerizes under UV illumination from the *trans* to the *cis* form, characterized by a higher steric hindrance [124]. This is the first example of MOF showing photoswitching for catalytic activity (activity in the dark, no activity under UV radiation) and catalytic selectivity (under UV reaction).

In another instance, under the same success, it has been evidenced by means of arylazopyrazole phosphonic acid molecules (*butyl-AAP*-C_18_PA) deposited on α-Al_2_O_3_(0001) single crystals, and the surface density has been shown using the Langmuir−Blodgett technique. This surface density facilitates large changes in molecular structure by applying time-dependent vibrational, namely sum-frequency generation (SFG), spectroscopy [144]. In this respect, nonlinear optical (NLO) responses and second harmonic generation (SHG) of photochromic molecules are welcome chemical structures in academic and industrial aspects [147].

### 4.2. Dithienylethene Photoswitchable Catalysts

**Figure 4:**
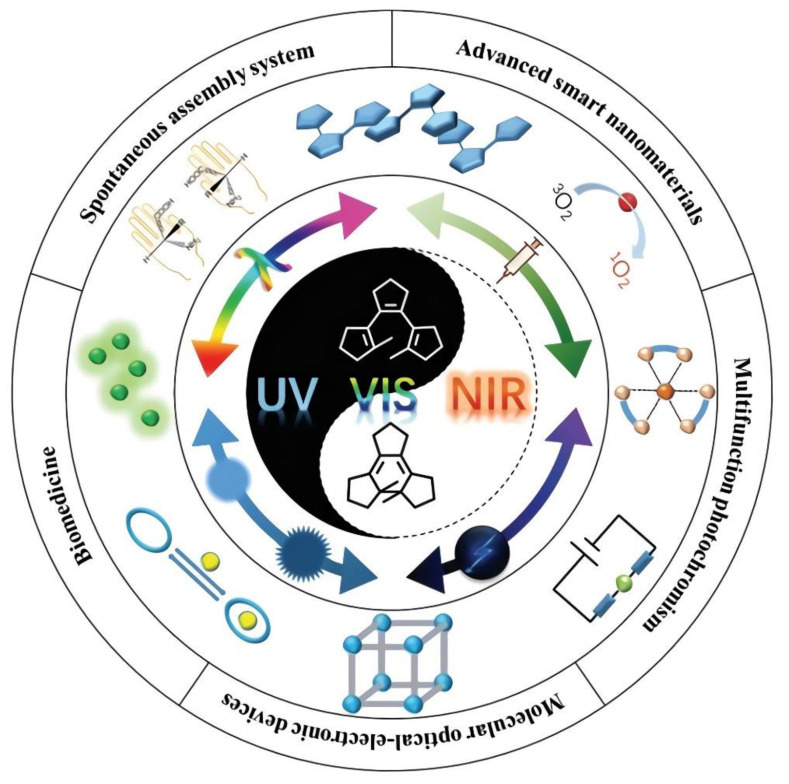
Diarylethene: a stimulus-responsive functional molecular switch. Reproduced with the permission of Wiley-VCH GmbH from [148]. Copyright 2022, John Wiley & Sons.

With the demand for photochromic molecules for the development of diarylethene (DAE) photoswitches, the aims have centered on the attention to finding an ideal DAE molecular switch, as objectively described by Cheng and co-workers [148]. Under light irradiation, diarylethenes (DAEs) undergo isomerization that is accompanied by innovative changes in their physical and chemical properties, as represented in Figure 4.

The dithienylethene (DTE) architecture contains two thiophene groups connected to a cyclopentene derivative at their 3- positions. Dithienylethenes (DTEs) are a rare class of small molecules capable of undergoing photochemical electrocyclization that is rapid, reversible, and thermally forbidden. The DTE scaffold possesses arguably the best photoswitchable characteristics of rapid isomerization, thermal irreversibility, and fatigue resistance, but also allows uses for photoredox reactions and energy-transfer modulators. DTE scaffolds can undergo a thermally forbidden but photochemically reversible 6π-electrocyclization. Catalytic ON/OFF capabilities arise from an extensive conjugation of rings that connect to the central core. This way, the performance of many ligands can be affected by the electron density at their binding sites. However, DTE-based systems still lack the distinct ON and OFF modes of electrical switches and are often prone to degradation.

Photoswitchable catalysts based on dithienylethene derivatives have been reviewed by Majee and Presolski [149]. DTEs are classified as P-type molecules (photochemically reversible only), T-type (thermally reversible) azobenzene, and spiropyran motifs [149]. In one attempt to control the stereochemical outcome of the catalyzed transformations, the ligands of DTE could create photoswitchable, chiral catalysts that induce enantioselectivity. In 2005, a photoswitchable DTE catalysis was reported by Sud and colleagues [150] based on a copper dithienylethene complex. Irradiation with 313 nm light leads to the formation of a deep-red, ring-closed isomer. It was hypothesized that the system’s rigidity affects the chelation so that the copper ion is no longer in a strongly chiral environment. In further work, Branda and colleagues [151] developed a reversible molecular switch that controls enzyme activity using a DTE system and Cu (II) iminodiacetate incorporating sulfonamide. It was observed that the flexible isomer could deliver the inhibitory sulfonamide group to the active site much better and simultaneously with the reversible binding of Cu to the histidine residues near the catalytic Zn (II) center. In such a study, the DTE molecule is not an accelerating ligand but an inhibitor of the catalyst human carbonic anhydrase I (hCAI).

A photoswitchable molecule that features a phenyl ring in place of one of the thiophenes in traditional DTEs was developed to undergo keto-enol tautomerization [152]. Ring-opening polymerization is photo-controlled, and thus the photoswitchable catalyst can regulate the relative incorporation of two different monomers. This system enables the in situ remote control over the ring-opening polymerization of l-lactide. It allows the incorporation of trimethylene carbonate and δ-valerolactone monomers in copolymerizations. The keto-enol tautomerism is controlled to reverse the hydrogen-bonding-mediated monomer activation of ON and OFF, representing a step toward accurate spatiotemporal control.

DTE-based photochromic catalysts can be modulated with light to act in a wide range of reactions. However, DTE scaffolds require more investigation to surpass the catalyzed transformations’ stereochemical outcome for all desirable applications. It can be outlined that DFT scaffolds are currently considered homogeneous catalysts that are investigated to achieve repeated quantitative photoconversion in both directions with irradiation times that are orders of magnitude shorter than the duration of the catalyzed reaction. It is important to note that a proper ON/OFF system might be currently impossible to achieve when using DTEs.

Noteworthily, in the same order of ideas, an excellent research work that uses donor (D) and acceptor (A) groups onto DTE allows one to vary photophysical properties. Here, the photochromic D-π-A type DTEs are used to investigate the relationship between their structure and photochemical properties. In the report implemented by Park et al., the donor groups 3,5-dimethoxyphenyl and 4,4′-tert-butyltriphenylamino, and benzoic and cyanoacetic acid as acceptors groups, were attached to an oxazole-based DTE core [153]. The authors detailed the photochromic properties of the designed compounds when exposed to UV (365 nm) and visible (>400 nm).

With the number of new technologies that are being released, an innovative study evaluates the embryotoxicity of DTE-modified peptides upon photoswitching using analogues based on the β-hairpin scaffold of the natural membranolytic peptide gramicidin S. Regarding the lethal dose of photoswitchable peptide, in vivo toxicity values concerning 50% lethal doses (LD_50_) could be compared with in vitro cytotoxicities using larvae of the model organism *Danio rerio* [154].

## 5. Future Perspectives

Nanotechnology and nano-modification offer new possibilities for significantly improved materials, and the importance is to create new nanostructured materials with photocommutable properties. One focus with respect to photoswitching materials is that they can be functionalized for very specific applications in expanded structural combinations either as azo compounds or as supramolecular systems. In this context, the constant development of assembly chemistry has provided a perspective beyond the molecular level for practical application, creating photochromic molecules from laboratory exploration to broad industrial areas. This is certainly encouraging and provides valuable insights for the new development of innovative structured materials.

The last point is that MOFs have been developed in various scientific fields and are functional in an infinity of essential industrial fields. From them have come various coordination-driven self-assembly structures such as MOPs, whose research field is less explored compared to MOFs and even less for the photoswitchable control of processes controlled by UV-Vis electromagnetic radiations. This is because only fourteen types of polyhedra have been discovered in MOP assembly. Evidently, this number is significantly lower than that of thousands of known MOF topologies. Moreover, the captivating symmetric topologies and the discrete structural features can limit the applications of MOPs, unlike MOFs.

Various scientific explorations have followed the same path to model other arrangements, such as the HOFs. A crucial part of the investigation of these materials is to realize perfectly designed symmetrical topologies with chemical stability, even under mechanical stress. We need to remember that symmetry is the essence of every chemical reaction in nature; here, the photonic is vital par excellence.

There are several examples of this, such as in the field of optical sensing biomimetic materials with the ability to incorporate structural color due to nanoarchitecture variants integrating regularity and irregularity. This phenomenon recreates patterns of hierarchically structured photonic crystals, which provide properties of coloration, camouflage, protection against radiation, and photosynthesis where symmetry plays an important role [155].

With great similarity, the polarization-sensitive emission features of the Luminescent lanthanide metal–organic framework (Ln-MOF) crystals with distinct PL spectra provide a desirable strategy for the polarization-tunable photonic. In the same context, using the Eu-BTC (1,3,5-benzenetricarboxylic acid, BTC) compound, high-capacity photonic barcodes have been designed by manipulating the photoluminescence (PL) spectra with the idea of being transformed into a specific recognition code to design novel photonic barcodes [156].

## Figures and Tables

**Table 1:** Literature overview on MOCs’ synthesis and applications.

Ref.	Metal Ion	Organic Ligand	Ligand Nature	Cage Structure	Geometry	Guest	Cage Containing Polymer	Irradiating Light λ (Reversible)	Light Exposure Time (min)	Reusability (Cycles)	Mechanism	Application
[61]	-	ABOEG	Chiral acyclic oligoethylene glycol	-	-	K^+^/Cs^+^	-	365/448 nm	30/30	-	*E/Z* isomerization of the azobenzene unit	Synthesis of optically active amines
[60]	Cu(II)	*m*-BDC	Coumarin based	Cu_24_L_24_	Cuboctahedra	-	PEG	368 nm	480	4, 12 states	Transition between Cu(II),Cu(I), and Cu(0) states	Catalysis of azide-alkyne cycloaddition (CuAAC) reactions/Sol–gel transition/Color switches
[64]	Pd(II)	Z1	Bidentate bispyridyl	Pd_2_L_4_	Square planar	TA		312/365 nm	-	-	Photochromic ligand isomer interconversion	Tuning guest binding affinity and selectivity properties of supramolecular coordination complexes
[65]	Cu(II)	H_6_TDPAT	Melamine based	-	Cuboctahedron/Truncated tetrahedron/Truncated octahedron	CO_2_/CS_2_	-	>400 nm	720	5	Cu(II)/Cu(I) transition	Incorporate CO2/CS2 and CF3 groups into value-added heterocycles
[62]	Zn(II)	SD	Tetrameric silsesquioxane and pyrazole functionalized with azobenzene	Zn_8_L	Octanuclear space craft-like	-		365/450 nm	10/2	-	*trans*/*cis* ligand isomerization	Light sensitive molecular pump
[66]	Pd(II)	-	Tetratopic N-donor	Pd_2_L_4_	Hexanuclear	-	PEG	456 nm	60	4	Reversible cleavage of the metal–ligand bond	Sol–gel transitioning
[67]	Ga	DAB	Polyaromatic bridge	Ga_4_L_6_	-	CA		400 nm	-		photoinduced electron transfer (PET): Ga4L6 12− cage absorbs photons and transfers an electron to the guest ion	Transfer energy to encapsulated guest molecules
[63]	Pd(II)	-	Bent bis-monodentate pyridyl	Pd_2_L_4_	-	*cis*-4,4′-AB	PEG	365 nm/white light	-	4	*cis*/*trans* isomerization of guest	Expelling the guest out of the cage/Spatially controlled lithographic deposition
[59]	Pd	IA	-	-	-	AAP	-	365/520 nm	5	10	*E* to *Z* isomerization of arylazopyrazole	Solubilization of E isomer of arylazopyrazole in water
[68]	Cu(II)	DPD	Azobenzene containing unit	Cu_12_L_24_	Cuboctahedra	MB	-	365/blue light	30	5	*trans*/*cis* isomerization of ligand	Expel the guest from the cavity
[69]	Pd(II)	DTE	-	Pd_2_L_4_	-	R/S CSA		313 nm	-		Interconversion between ligand open and photoisomeric closed forms	Expel the guest from the cavity

**Table 2:** Some azobenzene systems with photoswitchable characteristics.

Azo-Based Photoswitch	Substituent Groups	Substituent Configuration	Substituent Nature	$\lambda_{max}$ (nm) *trans* $\left(n\to \pi^{*}\right)$	$\lambda_{max}$ (nm) *trans* $\left(\pi \to \pi^{*}\right)$	Tethered Ligand	Ligand Configuration	Conformational Effect	Ref.
Tetra fluorinated azobenzene	Fluorine (4)	ortho	EWG	447	320	Iperoxo	para	Improve binding affinity to muscarinic receptor	[128]
MAG azobenzene	Fluorine (1)	ortho	EWG	473	365	Maleimide Glutamate	para	Control over activation of glutamate receptors	[129]
Azobenzene based polyamide	-NHCO-(2)	para	EDG	425	323	-	-	Decrease in the thermal half-life of the *cis* isomer	[130]
Azo-combretastatin	Methoxy (3) Ethoxy (1)	meta (2) para (1) meta (1)	EWG	-	<400	Ethoxy	para	Improve binding affinity to Tubulin through colchicine pocket	[131]
Diazocine	Acetamide derivative	-	-	-	<400	-	-	Inversion of conventional *cis* active state to a trans active state	[132]
APA	-	-	-	450	335	PEG	para	Control over polymer phase transition	[133]
Azo-AA monomer	-	-	-	>436	>365	4-phenylazophenyl acrylate-DMF	para	Up-shift in the phase transition temperature	[134]
Azobenzene based monomer	CH_3_	para	EDG	450	350	HOC_6_H_12_O	para	Solid to liquid transition of the azopolymer	[135]
Azobenzene-Schiff base	-	-	EWG	365	500	Al(III)-Cl substituted Salicylaldehyde derivative Schiff base	para-	Ring opening polymerization ε-caprolactone	[120]
Tri-azo substituted phosphine	Phosphorus	para	EDG	345	-	Ruthenium complexes	-	H generation by hydrolytic decomposition of AB	[136]
PLP–photoswitch–imidazole triad	PLP	meta	EWG	431	319	Imidazole	meta	Conversion of L-amino acids toD-isomers	[137]
Azopeptide catalysts	Pmh	meta (2)	-	448	-	-	-	Acetylation ofsugars	[138]
Azo-GFGH	Cyclodextrin	-	-	-	324	GFGH	-	Hydrolysis of 4-nitrophenylacetate	[139]
Azo-GFGH	Histidine residue	*-*	-	-	325	GFGH	-	Hydrolysis of p-NPA	[140]

## Data Availability

Not applicable.
